# The Role of Magnetic Resonance Imaging and Computed Tomography in Spinal Cord Injury

**DOI:** 10.3390/life13081680

**Published:** 2023-08-03

**Authors:** Omar Hussain, Mayank Kaushal, Nitin Agarwal, Shekar Kurpad, Saman Shabani

**Affiliations:** 1 Department of Neurological Surgery, Medical College of Wisconsin, Milwaukee, WI 53226, USA; omhussain@mcw.edu (O.H.); mkaushal@mcw.edu (M.K.); skurpad@mcw.edu (S.K.); 2 Department of Neurological Surgery, University of Pittsburgh, Pittsburgh, PA 15213, USA; nitin.agarwal@upmc.edu

**Keywords:** diagnostic imaging, magnetic resonance imaging, computed tomography, spinal cord injury, spinal trauma

## Abstract

Traumatic injuries of the spine are associated with long-term morbidity and mortality. Timely diagnosis and appropriate management of mechanical instability and spinal cord injury are important to prevent further neurologic deterioration. Spine surgeons require an understanding of the essential imaging techniques concerning the diagnosis, management, and prognosis of spinal cord injury. We present a review in the role of computed tomography (CT) including advancements in multidetector CT (MDCT), dual energy CT (DECT), and photon counting CT, and how it relates to spinal trauma. We also review magnetic resonance imaging (MRI) and some of the developed MRI based classifications for prognosticating the severity and outcome of spinal cord injury, such as diffusion weighted imaging (DWI), diffusion tractography (DTI), functional MRI (fMRI), and perfusion MRI.

## 1. Introduction

Spinal trauma can result in spinal column and/or spinal cord injury with the risk of developing devastating neurological damage. This can include paraplegia, quadriplegia, or even death. Blunt trauma, especially from motor vehicle accidents, is the most common cause of the injury, followed by falls, assault, and sport accidents [1]. Penetrating injuries compromise a much smaller subset of these injuries. Spinal injury tends to follow a bimodal age distribution with the first peak observed in adolescents and young adults, and the second peak seen in adults above the age of 65. Injuries to the cervical spine are more common than thoracolumbar due to greater mobility in the cervical spine [1].

Imaging plays an important role in the initial phase of assessment of patients with possible spinal trauma. The choice of imaging modality depends on the clinical presentation and availability of imaging resources at the trauma center. Currently, computed tomography (CT) and magnetic resonance imaging (MRI) are the most utilized imaging modalities [2]. Further, there are a number of newer imaging technologies such as advancements in computed tomography (CT), diffusion tensor imaging (DTI), functional magnetic resonance imaging (FMRI), and perfusion-based imaging. Of these, DTI has been studied the most.

In this paper, we will discuss the role of CT and MRI in the diagnosis and prognostication of SCI and highlight recent developments, promising new techniques, and their potential clinical impact.

## 2. Computed Tomography (CT)

CT imaging is possible through the use of X-ray beams that pass through the body and are captured onto a moving set of detectors [3]. The captured images are then processed and can then be viewed in three dimensions in the axial, coronal, and sagittal planes. These three-dimensional reconstructions allow spinal injuries to be easily characterized [4]. Advancements in computation and technology have allowed the newest generation of CT scanners to capture high resolution images with slices less than 1 mm thin [5]. Multidetector computed tomography (MDCT), with arrays that can have up to 256 detectors, is the preferred imaging modality for the initial evaluation of patients with suspected spinal trauma [4].

**Figure 1:**
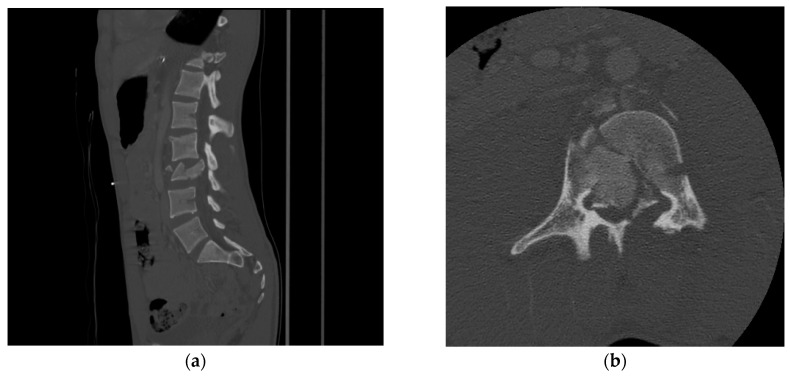
(**a**) Sagittal and (**b**) axial view of CT showing L3 burst fracture with significant retropulsion of the vertebral body into the spinal canal and loss of body heigh. There is involvement of the posterior elements.

**Figure 2:**
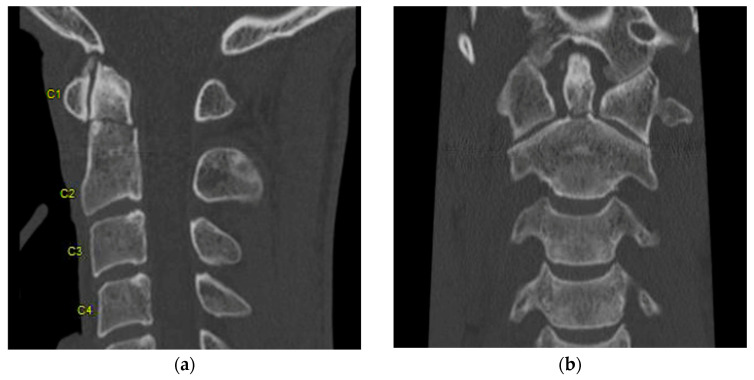
(**a**) Sagittal and (**b**) coronal view of CT showing type II dens fracture. “C1–C4” Designate each corresponding spinal level.

The American College of Radiology recommends CT as the initial imaging modality for the evaluation of spinal trauma in adults and children older than 14 years [6]. CT provides great detail regarding osseous anatomy and its associated pathology (Figure 1 and Figure 2) but can also reveal significant abnormalities such as traumatic disk herniations and epidural or subdural hematomas in the spinal canal.

The higher sensitivity of CT compared to plain film radiographs in detecting fractures has increased the diagnostic accuracy and offset concerns about the increased risk from added radiation exposure. CT has a sensitivity of 97–100% in identifying cervical spine fractures compared to cross table lateral X-rays, which have a sensitivity of only 63% [7]. In thoracolumbar trauma, CT was associated with sensitivity ranging from 78.1 to 100%, while plain radiographs had sensitivity in the range of 32–74% [8]. Despite the increased cost of operating and performing a CT scan over X-ray, it was found that in the setting of urban trauma centers, CT scans were cheaper than X-rays [9]. This is because Glover et al. took into account the cost of hospital settlements for injuries that were missed on X-rays, which significantly increased the cost associated with plain radiographs [9].

In addition to providing details in bony anatomy, CT scans can reveal significant soft tissue abnormalities such as traumatic disk herniations and significant epidural or subdural hematomas in the spinal canal. The trend toward usage of CT as the primary imaging modality was shown in a recent study by Shabani et al. [2]. They conducted a survey across the six AO regions around the world, and it was determined that in neurologically intact patients with spinal trauma the predominant imaging modality for all AO regions was CT [2].

In patients with abnormal anatomy, either congenital or iatrogenic, CT remains important in understanding this anatomy, in anticipation of surgical intervention. For example, in patients with Ankylosing Spondylitis (AS) or diffuse idiopathic skeletal hyperostosis (DISH), the CT scan will show regions of bony fusion with greater detail than X-ray or MRI [10]. In a similar fashion, in patients with metallic spinal hardware and concomitant suspected spinal trauma, CT is the best methodology to evaluate the altered bony anatomy and evaluate the hardware itself [11].

In addition to standard non-contrast CT imaging, CT myelography has played a small role in diagnosing spinal cord injury. CT myelography involves an intrathecal injection of iodinated contrast via lumbar puncture and subsequent CT scan through the spine to identify areas of opacification or lack thereof, suggesting stenosis [12]. With the advent of MRI, the indications for CT myelography are becoming more limited, and its role in diagnosing acute spinal cord injury is essentially no longer applicable. In patients who MRI is contraindicated for, CT myelography may assist in operative planning [12]. The time and invasive nature of this technique make it less commonly used in the acute or emergent setting.

Newer technological advances with CT include dual energy CT (DECT) and photon counting CT. Photon counting CT directly measures each photon as it hits a semi-conductor detector, as opposed to standard CT detectors which use a scintillator to generate a visible light when hit by an X-ray that is then detected by a photodiode [13]. Currently, there are no reports in the literature that describe photon counting CT technology with respect to evaluating spinal trauma; however, this is certainly an area of future study. Dual energy CT (DECT) uses photons at two different energy levels and then analyzes the differences in absorption at these energy levels [14]. A common use for DECT within spinal pathology is through metal artifact reduction, and bone marrow edema identification [15]. In patients with prior spinal hardware, the bone and soft tissue around the metal hardware can be hard to visualize due to the dark or bright stripes projecting from the metal. DECT can increase the energy level to attenuate the artifact from the metal, while also generating an image that maintains enough detail for anatomic considerations [15].

For patients being evaluated in the trauma setting with CT, fractures can be missed, confused as vascular channels within the bone trabeculae, or vice versa. In these settings, MRI would be used as the gold standard to determine the presence of bone marrow and/or soft tissue edema, which would help to identify a vertebral fracture. MRI is longer, costly, and may not show bone morphology too well compared to standard CT [16]. DECT is able to demonstrate bone marrow edema within vertebral fractures, and delineate acute from chronic fractures, with a sensitivity of 86.2%, specificity of 91.2%, and an accuracy of 89.3% [16,17,18]. DECT remains a promising avenue for spinal trauma evaluation; however, it is still limited in that it cannot image the spinal cord with any detail.

**Figure 3:**
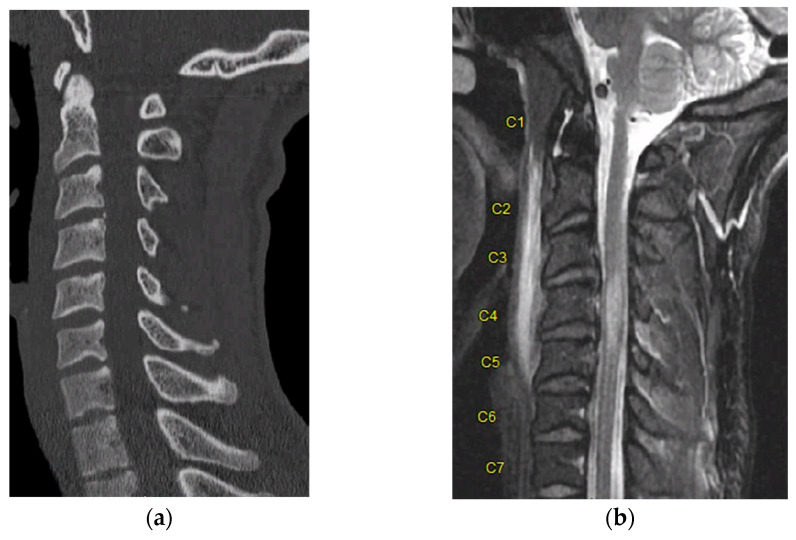
(**a**) Sagittal CT demonstrating no bony injury or malalignment to a trauma patient. (**b**) Sagittal MRI STIR sequence demonstrating cord signal change with evidence of contusion spanning from C3–C5. “C1–C7” designate each corresponding spinal level.

One such example that highlights this comes from our own institution. An adult male patient with no past medical history was brought into our emergency department with negative CT of the entire spine after a rollover motor vehicle crash. Upon initial evaluation, he was displaying clinical signs of spinal cord injury including weakness of the bilateral lower extremities. Thus, MRI was obtained, which demonstrated ligamentous injury and cord signal change (STIR signal) spanning from C3–5 (Figure 3).

This highlights how CT imaging is not without problems, and additionally, that artifacts from image acquisition can occur, such as metal, beam hardening, scatter, and ring artifacts [19]. A combination of advancements in mathematics and technology has allowed for these artefacts to be reduced. For example, photon counting CT has been shown to reduce beam hardening artefact while increasing spatial resolution [13,19]. Additionally, CT does not determine the severity of spinal cord injury, nor does it assist in assessing the prognostication of the injury.

## 3. Magnetic Resonance Imaging (MRI)

**Figure 4:**
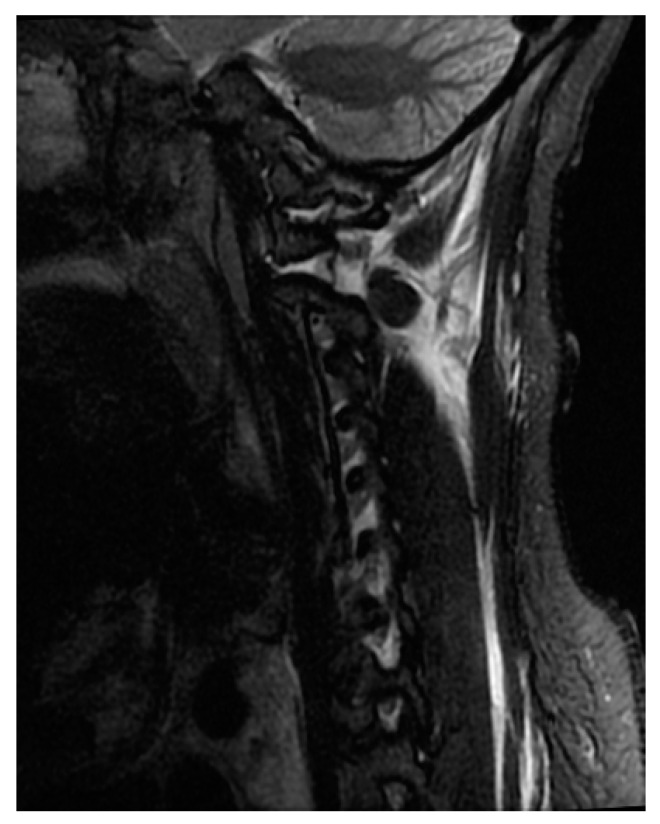
Sagittal view of STIR sequence of MRI shows signal changes in the C1/2 and C1/condyle joint.

**Figure 5:**
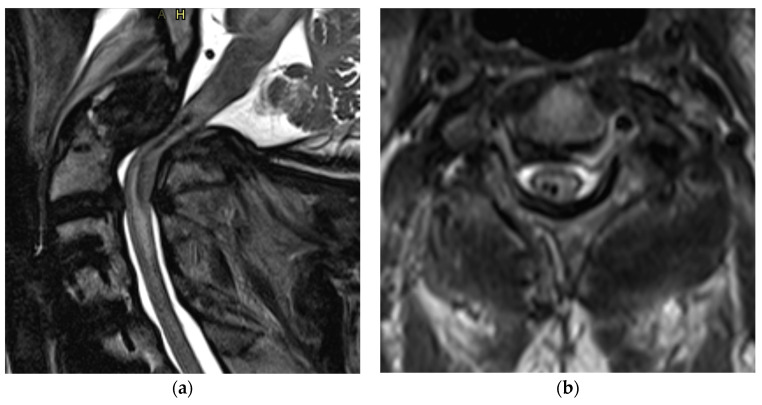
(**a**) Sagittal and (**b**) axial view of T2 MRI showing severe spinal cord injury with intramedullary hemorrhage.

MRI is currently considered the gold standard imaging modality for the evaluation of patients with spinal cord injury and spinal trauma [20,21,22]. MRI has multiplanar capabilities with high-contrast resolution and is, therefore, capable of providing information regarding spinal cord compression, ligamentous instability (Figure 4), disk herniation, contusion, and hemorrhage (Figure 5), as well as injury to vertebral bodies and paraspinal tissues [23,24,25]. In a review done by Sliker et al. (2005) in patients with blunt trauma, the authors reported 22.7% ligamentous injury detection by MRI, of which 80.8% required treatment [26]. Despite the time taken to perform MRI, it is still recommended in the pre- and post-operative stages when feasible [27]. Not only is MRI noted to be safe, when protocols are followed, it also directly impacts clinical decisions for the surgeon [27].

MRI sequences recommended for spinal cord trauma include sagittal fast spin-echo (FSE), axial and sagittal T1-weighted imaging (T1W), axial and sagittal FSE T2-weighted imaging (T2W), sagittal T2-weighted fat-suppressed imaging, and axial T2*-weighted gradient echo imaging. The variability in MRI recommendations depends on the clinical presentation and/or MR center capabilities.

Conventional MRI, both T1- and T2-weighted imaging, have limited prognostic value. Currently, T2-weighted imaging, as well as the short tau inversion recovery (STIR) sequence, are the most important sequences in acute spinal cord injury since they have high sensitivity to intramedullary pathology, and to acute injury/edema [28,29]. STIR images are sensitive in detecting edema within the spinal cord as well as the surrounding soft tissue and ligaments, with the advantage of providing more consistent fat suppression compared to fat-suppressed T2WI [30]. The presence of STIR signal within the spinal cord in combination with acute neurologic deficit and possible bony or ligamentous injury should be factors that aid in decision making for the spine surgeon [27].

T2-weighted MRI is sensitive to the paramagnetic effects of iron present in blood products. Intramedullary hemorrhage presents as an area of hypo-intensity on T2W imaging, and its presence has a prognostic implication (Figure 4) [28,31,32]. T2*W and susceptibility-weighted imaging (SWI) sequences are also used for the detection of intramedullary hemorrhage [33].

Different classification systems for determining the extent of neurological injury have been created based on conventional MRI characteristics and findings (Table 1). The first classification system was created by Kulkarni et al. [34]. They determined that spinal cord hemorrhage, edema, and swelling were associated with spinal cord injury (Table 1). Bondurant and associates’ classification (Table 1) divides the MRI signal pattern into four subtypes based on modification of the Kulkarni et al. classification system [23]. Schaefer et al. further characterized four types of MRI findings that were associated with spinal cord injury. Talbot and colleagues developed a novel five-point ordinal MRI score (BASIC score) which was predictive of neurologic impairment [29,35,36].

The above classifications were used to prognosticate recovery in patients with SCI. In the study by Schaefer and associates, Type 3 patterns were associated with greater recovery compared to Type 1 and Type 2 patterns [37]. Similarly, Flanders et al. showed that the presence of hemorrhage and length of edema on MRI were predictive of neurological improvement [31]. In another study done by the same group, the longer segments of edema and hemorrhage correlated with poor functional recovery [38]. In a prospective analysis, Boldin and colleagues noted that hemorrhagic lesions of 4 mm or less were associated with incomplete SCI and neurological recovery [39]. However, a hemorrhage greater than 10 mm—particularly in the cervical spine cord—was often associated with complete SCI [40]. 

The main drawback of MRI imaging is the time taken to acquire the scans. However, there are protocols to mitigate this. A T2 sagittal survey can be performed with the remaining sequences done only through the level of interest identified on sagittal survey. This has significantly decreased scan acquisition time in many centers. Constant communication of the MRI technician with the radiologist and spine surgeon is important to ensure these focused exams are performed efficiently.

## 4. Diffusion Weighted Imaging (DWI)

Although conventional MRI has been used for prognosis and determining the extent of neurological deficit, it still lacks the specificity to be used as a biomarker. Across some centers, diffusion weighted imaging is being incorporated into the imaging of patients with SCI. The hope is to have a better understanding of the microstructural changes in the spinal cord as a result of injury, and then to ultimately improve quantitative indicators of prognosis [41]. Diffusion tensor imaging (DTI) is an extension of DWI. It measures the direction of water diffusion inside tissues. In axons, water diffusion is limited by the cell membrane and myelin sheath which results in high diffusion in the direction parallel to the white matter tract and low diffusion perpendicular to it.

Several diffusion metrics are used to characterize the diffusion of water molecules after spinal cord injury, with the principal metrics being fractional anisotropy (FA), mean diffusivity (MD), radial diffusivity (RD) or transverse apparent diffusion coefficient, and axial diffusivity (AD) or longitudinal ADC (lADC). The clinical application of DWI/DTI to evaluate SCI in humans has stemmed from earlier contributions derived from experimentally induced SCI in animal models, given the similarity of mechanism involved in inducing cord injury via mechanical forces in both humans and animals. An exploration of studies highlights several metrics used for describing the changes to diffusion in the spinal cord white matter, with some of the earliest work documenting alterations to diffusion in the injured cord both parallel and perpendicular to the white matter tracts running through the spinal cord. This was observed in the backdrop of a normal appearing spinal cord on conventional MR imaging. In the work by Loy et al., quantifiable changes to diffusion allowed for the determination of the severity of injury. Furthermore, the changes in the diffusion metric of AD were found to be correlated with recovery of locomotor function. Another study showed reduction in FA to be time dependent post-injury. These inferences of the animal studies pointed to the potential of DWI/DTI in the prognostication of outcomes following SCI while highlighting which metrics might be the most consistent in evaluating white matter disruptions in the spinal cord following injury.

Fractional anisotropy (FA) and mean diffusivity (MD) are the two most frequently used measurements within DTI. DTI metrics were shown to have significant relationships with acute motor scores [42] and long-term neurological outcomes in cervical SCI [43]. Shabani et al. (2019) evaluated the diagnostic and prognostic value of DTI in both cervical and thoracic SCI. In patients with cervical SCI, FA in the high cervical region (C1–2) correlated significantly with injury severity and neurological recovery one-year post-injury. In patients with thoracic SCI, no significant correlation was seen between FA and injury severity at one-year follow-up. The distance between the measurement site for FA (C1–2) and the location of the injury in the thoracic spine may contribute to this finding [44].

A study by Poplawski et al. found significant reductions in FA and elevations in MD, and radial diffusivity (RD) proximal to the injury site (within one level above and below the epicenter). DTI indices rostral to the anatomic level of the injury consistently measured immediately showed better correlation with the extent of injury and with accuracy in predicting neurological recovery at six months than indices measured at the epicenter. FA and RD were measured one level rostral to the injury site had the best sensitivity and specificity for predicting the severity of injury. However, MD offered the best prediction of neurological recovery [45]. 

The usage of DTI in the clinical setting has been limited, although DTI has shown promise as a marker of SCI damage and as a prognostic marker. A low signal to noise ratio and increased artifacts due to cardiac and respiratory motion make visualization of the spinal cord boundary challenging. This results in difficulty in the quantification of DTI parameters. Among centers, there are wide variations in scanner performance, protocols, and post-process techniques, which makes generalizability of DTI findings difficult. Furthermore, the added time and resources required for the post-processing and computation analysis limit clinical use in the acute setting.

While acquiring DWI images, several assumptions are made by the mathematical processing programs. Such assumptions include the magnetic field having uniform homogeneity and the pulses generated from radiofrequency coils being perfectly shaped. The current technology limits these assumptions from being truly met and, as a result, the images generated tend to be less accurate or lower in quality. Image artifacts can occur, given the gradient magnitudes and switch rates achieved with the hardware being used. Furthermore, the use of echo planer imaging (EPI) sequence, which allows for a drastic shortening in imaging acquisition time and reduces the impact of motion artifact on image acquisition, has the downside of low spatial resolution and poor signal to noise ratio (SNR) [46]. The MR scanners with higher Tesla magnets in recent times have led to the susceptibility artifacts being more pronounced compared to images generated by scanners with lower Tesla magnets. Another concern with DWI is inconsistency in the reproduction of the ADC values that stem from the rapid transition between on and off states of the diffusion gradients that cause distortions to the image quality [47].

Recent advances to overcome the limitations of DTI have also emerged. DTI notably is confounded by edema which can mask the injury to neurons. Skinner et al. demonstrated in animal models a DWI technique to reduce the added signal from edema, termed double diffusion encoding (DDE) or filtered DWI (fDWI) [48,49]. It uses diffusion weighting to suppress signals from extracellular water such as edema and CSF, which minimizes their effects on the DWI metrics. This method has shown to provide better contrast than DTI and had strong associations with the functional outcomes in a rat model of SCI. Recently, it was demonstrated that the fDWI technique is compatible with clinical DWI methods (i.e., pulse sequences) with only minor modifications to the applied diffusion directions specific to the spinal cord anatomy. fDWI has a faster acquisition, and the process of data analysis is fully automated which allows for broader evaluations across different sites and institutions. Another factor that impacts the precision of the diffusion measurements is spatial in-homogeneity of the diffusion gradients. BSD-DTI is a newer method in DTI described by Kryzak and Olekniczak [50]. It involves using precisely defined anisotropic phantoms that maintain their properties throughout measurements. By employing the same imaging sequence for both calibration and imaging, the method eliminates sequence-dependent errors. Several analyses have confirmed the effectiveness of this calibration method first in vitro and later in vivo. Unlike the standard method that relies on precise knowledge of diffusion gradient characteristics, BSD-DTI directly measures the space-dependent elements of the b matrix. This is also not without error, and Kyrzak and Olekniczak describe the development of better phantoms to the reduce signal to noise ratio [50]. Understanding the attempts at reducing error is critical in clinical practice, as the tracts within the spinal cord are tightly packed together within a relatively small space. Translation of these methods into clinical use for patients with SCI is under investigation.

## 5. Functional MRI (fMRI)

Functional MRI (fMRI) is an advanced imaging technique that is more commonly utilized in the brain, however its utilization in evaluating spinal cord pathology is becoming more recognized. Functional MRI takes advantage of the increased oxygen metabolism of more active neurons, and is able to identify a blood oxygen level dependent (BOLD) signal which monitors changes in deoxyhemoglobin concentration in any given voxel during a task given to the patient, or in resting state (rs-fMRI) [9]. Resting state fMRI helps by acting as a measure of the baseline connections between regions of the brain and spine. This can further characterize networks of fibers that may continue to exist post-injury. Task-related fMRI studies identified by Freund et al. in 2019 have demonstrated that there is spinal activity retained in response to stimuli, both above and below the site of injury. This suggests there are retained networks that may be re-engaged to promote recovery. It also may explain the presence of post-SCI associated neuropathic pain [51]. 

In order to understand the use of fMRI in SCI, it is first important to characterize the normal state in the healthy population. Many studies sought to establish this normal baseline of fMRI in the spinal cord, both in resting and task-based situations. Landelle et al. conducted a meta-analysis in 2021 to characterize the activation of both sensory and motor pathways within the spinal cord, confirming what is already known about the paths these two pathways take [52]. 

A meta-analysis from 2016 identified eight previous fMRI studies relating to spinal cord pathology, with two regarding chronic spinal cord injury (cSCI). They found that patients with complete SCI were found to have decreased ipsilateral dorsal activation from the site of stimulus but increased bilateral ventral activation, as well as increased activity in patients with incomplete cSCI in the dermatome of normal sensation [53]. The reliability of these data was low however, due to the noted high risk of bias, liberal statistical methods, and the variation in protocols which attempted to increase signal to noise ratio. This demonstrates how early the field is in exploring the utilization of fMRI, as the body of literature grows and more reliable patterns of activation can be detected, fMRI may be able to identify key regions of the spinal cord that are still amenable to future regenerative therapies.

## 6. Perfusion MRI

More functional assessments of the central nervous system have further utilized MRI with perfusion-based imaging. MRI perfusion tracks blood flow to a selected tissue being imaged, either with a contrast agent, or via arterial spin labeling [54]. This is used to derive parameters similar to those seen with CT perfusion, such as blood volume, mean transit time, and blood flow. These parameters are frequently assessed for ischemia and penumbra within the brain. Spinal cord perfusion studies have more commonly been around to evaluate tumors. More research is bringing the use of perfusion MRI into the study SCI. Given that current treatment targets of avoiding hypotension are seeking to increase the perfusion of spinal cord tissue to increase the functional recovery of a patient, having a non-invasive imaging modality to monitor the progress of these therapies is vital.

There can be multiple mechanisms which disrupt spinal cord perfusion and blood flow, via direct vascular damage, disruption of sympathetic tone, or via edema within the cord, which will naturally lead to a diverse pattern of spinal cord blood flow. Similar to functional MRI, perfusion MRI has wide variability in the protocols used and these are not optimized for evaluating perfusion within the spinal cord itself. 

Meyer et al. used a rat model of cervical spinal cord injury and successfully identified a protocol that provided clear assessment of spinal cord blood flow without significant artifact [55]. After induction of acute contusion to the cervical spine in rats, the authors found a reduction in blood flow at the site of injury as well as an increase in transit times compared to non-injured tissue. The authors also noted that the area of reduced blood flow was generally smaller than the area of edema that is found on conventional T2 weighted MRI. This highlights that MR perfusion imaging generates unique data that can both aid in monitoring the initial injury and potentially serve as a prognostic marker for recovery.

Meyer et al. went on to trend imaging changes over time after spinal cord injury in a rat model, at 4 h, 48-h, and 12-weeks post-injury [56]. They concluded that the imaging findings are dynamic. Early spinal cord blood flow (SCBF) deficit volumes at 4 h were larger than the DWI lesion and were more correlated to the motor function at 12 weeks. Later imaging at 48 h demonstrated the opposite. At 48 h, the deficit from SCBF partially recovered, the DWI lesion increased in size, and the DWI lesion size was correlated with motor function at 12 weeks. It should be noted, however, that the SCBF deficit volume noted at 4 h was a better predictor of long-term functional outcome [56]. 

A similar model of spinal cord injury was induced in the thoracic spine of rats, and studies were conducted to correlate imaging findings with severity of clinical injury. Lee et al. demonstrated that spinal cord blood flow is also reduced after a thoracic cord injury, similar to the cervical cord. While there is a correlation with decreased spinal cord blood flow, DWI and particularly T1 weighted imaging carried a stronger and more consistent relationship with injury severity [57]. 

Future studies should explore how current management strategies such as increasing blood pressure and avoiding hypotension after SCI alter the SCBF deficit and how these correlate with motor outcomes.

## 7. Conclusions

In the management of patients with spinal trauma, imaging has an important role. CT is the first line imaging modality due to its fast and easy acquisition and high sensitivity to detect bony fractures. Emerging technologies within the field of CT, particularly DECT and photon counting CT, have been able to increase the sensitivity for detecting spinal trauma. MRI provides detailed information regarding ligaments, soft tissues, disk, and spinal cord. Conventional MRI can depict the location and severity of injury to the spinal cord, but it lacks the capability to provide any information about its microstructure. Some classification systems that identify key findings within MRI exist and provide a basis for some prognostication after spinal cord injury. Diffusion tensor imaging provides information about spinal cord microstructure, but its clinical use is limited by sensitivity to artifact, scanner variability, and the need for extensive post-processing of images. FDWI was recently developed to overcome some of the DTI limitations, but it is in the early stage of development. Functional MRI can aid in identifying tracts or networks in patients after SCI that may end up as therapeutic targets in the future. Perfusion MRI tracks spinal cord blood flow after injury and can identify regions within the cord that have early blood flow deficits, which have been found to be correlated significantly with long term motor outcomes. While it is important to understand the function and utilization of each imaging modality as a standalone tool, the key is being able to make clinical decisions based on information gathered and synthesized from all the available imaging techniques. Substantial research to validate the most optimal protocol for running these imaging modalities has yet to be conducted. Once validated, these technologies can be used to both advance pre-clinical therapeutic target research and drive adoption in high volume spinal cord injury centers. Advanced imaging techniques may be in the early stages of research but the growing body of evidence is readily allowing the care of spinal cord injury patients to be improved in the long run. 

## Figures and Tables

**Table 1:** MRI based injury classification schemes.

Classification Scheme	Type	Name
Kulkarni et al. (1987) [34] MRI patterns for prognosticating acute spinal cord injury	Pattern 1	Cord hemorrhage
	Pattern 2	Cord edema
	Pattern 3	Mixed
Bondurant et al. (1990) [23] classification scheme for prognostication of acute spinal cord injury	Pattern 1	Normal MRI signal
	Pattern 2	Single level edema
	Pattern 3	Multi-level edema
	Pattern 4	Mixed hemorrhage and edema
Schaefer et al. (1992) [37] types of MRI findings associated with acute spinal cord injury	Type 1	Central intramedullary cord hemorrhage
	Type 2	T2 hyperintense contusion extending longitudinally greater than 1 vertebral body
	Type 3	T2 hyperintense contusion, confined to a single vertebral body
	Type 4	No evidence of SCI on MRI
The Brain and Spinal Injury Center Score (BASIC) for classifying acute SCIs on the basis of axial T2-weighted imaging	Basic 0	No intramedullary cord signal abnormality
	Basic 1	Intramedullary T2 hyperintensity confined to central gray matter
	Basic 2	Intramedullary T2 hyperintensity involves both gray and white matter but does not cover the entire transverse extent of the spinal cord
	Basic 3	Intramedullary T2 hyperintensity covers the entire transverse extent of the spinal cord
	Basic 4	Intramedullary T2 hyperintensity covers the entire transverse extent of the spinal cord plus T2 hypointense foci

## Data Availability

Not applicable.
